# Generating "Différance" or an Ontology That Is Same Old Same Old: Comment on "The Generative Mechanisms of Financial Strain and Financial Well-Being: A Critical Realist Analysis of Ideology and Difference"

**DOI:** 10.34172/ijhpm.8565

**Published:** 2024-08-19

**Authors:** Lynn McIntyre, Catherine L. Mah

**Affiliations:** ^1^Department of Community Health Sciences, Cumming School of Medicine, University of Calgary, Calgary, AB, Canada.; ^2^School of Health Administration, Faculty of Health, Dalhousie University, Halifax, NS, Canada.

**Keywords:** Pragmatism, Public Health, Equity, Ontology, Pandemic, Ideology

## Abstract

Glenn and colleagues carefully conducted a realist review of initiatives introduced in high-income countries intended to improve financial well-being (FWB) or reduce financial strain (FS) during the early days of the pandemic. They found that these initiatives were underpinned by either neoliberal or social equity ideologies, within which, social location acted on different groups. In this commentary, we suggest caution in applying labels such as neoliberalism and social equity when lumping social welfare policies; labour policies; housing and financial services policies; and service provision for health, seniors, childcare, and education across welfare state regimes. We also caution against aggregating equity-deserving groups from different contexts into a single otherness. We suggest a pragmatic reinterpretation of the study’s findings and in future examinations of post-pandemic recovery in accordance with long-standing pragmatic methods of working in public health that seek to improve population health and well-being through collective action.

 Written during the depths of the SARS-CoV-2 pandemic, Glenn and colleagues assembled a realist rapid review of English-language academic and practice-sourced literatures on initiatives introduced in high income countries intended to improve financial well-being (FWB) or reduce financial strain (FS).^1^ The objective of the review was to identify FWB/FS initiatives reported during the 2015-2020 period that worked, for whom, and under which circumstances, to inform pandemic recovery and policy responses. From the vantage of 2024, our task is to interrogate their certainties of 2021.

 Glenn and colleagues’ consideration of 75 documents on FWB/FS initiatives led them to group them into those underpinned by a: (1) neoliberal ideology; or (2) social equity ideology, with a cross-cutting social location mechanism of action on different groups. Glenn et al conclude with disquiet that governments’ responses to the early days of the financial shock caused by pandemic shutdowns, when the cultural mantra was “we are in this together,” had already begun to shift to “getting back to work.”^1^ This is an appealing analysis aligned with the dissipation of ‘building back better’ which was the original recovery plan.

 Our reflection on this paper concerned with an analysis of ideology and difference led us to Jacques Derrida’s 1963 coinage of the term “différance” because his lens of deconstruction examines both differences in meaning, and how the act of inscribing introduces a deferral of meaning.^2^ We suggest that the empirical data of the realist review could be construed to identify different causal mechanisms, and that the totalized meaning garnered from the study’s short timeframe, with hindsight, should have been deferred to the pragmatic.

## Three Innovations

 Glenn and colleagues’ carefully conducted study was based on innovative concepts that are often overlooked in systematic or scoping reviews and incorporated three innovations. First, they chose novel outcomes for health and public policy work—FS (the negative) and FWB (the positive). Their work on these two constructs has been accepted by public health journals^3^ because it adds dimensionality to income as a determinant of health. Just as subjective well-being and perceived social status add experiential dimensionality to health status and socioeconomic status respectively, FS and FWB require individual interpretation—an agentic dimension within power relations.^4^ That is a strength when a more precision public health approach is desired but may be a weakness when an improvement in population health outcomes is sought.

 The second innovative concept employed in the paper was their choice of critical realism to underpin their rapid review approach. The justification was to answer the core questions of a critical realist review—What works, under what circumstances, and for whom?—in order to uncover what realists refer to as the ‘generative mechanisms’ for relational phenomena.^5^ The examination of FS or FWB in relation to initiatives needs to expose these relational phenomena. In the context of population health science, realists’ goal in uncovering generative mechanisms is to examine deep causes underpinning health, including those comprising social reality “as it (naturally) exists,” and in all its complexity.^5^ Defined by Glenn et al as the “underlying drivers of outcomes in real-word settings,”^1^ other public health readers might recognize that generative mechanisms bear some resemblance to Geoffrey Rose’s causes of the causes. We are most familiar with this idea now as root causes but it is applicable to newer studies of social and structural determinants of health. But when Glenn et al describe their analysis framework as context-mechanism-outcome, generative mechanisms are further explicated as “the underlying architecture that comprises regularities or trends.”^6^ This is an important turn as architecture is a metaphor for structure and mechanism implies a process.

 As such, it can be interpreted that Glenn et al have conducted their review on the basis of observations, positive, but counter-positivist,^6^ about the included FWB/FS initiatives set in relation to individuals’ varied freedoms that limit their capacity to actualize FWB within the structuration of ideology.^7^ Dominant here is “neoliberalism” but they consider “social equity” as well. By having to draw from literature from 2015 to 2020, Glenn and colleagues’ analysis is incomplete, missing most of the emergent pandemic sociopolitical and cultural “open system,” to use the critical realist concept,^8^ that they aim to analyze here as independent variables. One problem of agency (FS/FWB) in realism^7^ is that it misses such dynamic events as intensifying inequality and escalating political polarization, being but two of such pandemic system developments.^9^

 The third innovation in the paper was the integration of a social-equity analysis, namely, expanding upon the rapid realist review methodology to ask: What are the implications for equity? and included as part of the equity analysis, Why? But, if social location is understood to be the social position an individual holds within their society based upon social characteristics deemed to be important by any given society, then it could well be the generative mechanism of subjective well-being and perceived social status, but that is not necessarily helpful if FS and FWB are thought to be distinct from subjective well-being and perceived social status. Furthermore, the paper’s focus on “equitable actions” falls short of an equity analysis and does not answer the question, Why? Similarly, many material risks for the “equity deserving” are accounted for in their analysis, without having to come to terms with the nature of those same individuals’ and groups’ social (relational) alterity/otherness.^10^

## Discussion

 We do not presume to reprise the work of the paper’s peer reviewers nor do we intend to engage in theory fighting but we do intend to suggest caution. As members of the same team—people who care deeply about public health and social justice, we make the following observations and suggestions:

###  1. Same Old, Same Old

 While the practical findings under neoliberalism reveal some problematic policies and their effects on populations, the idea that they are “lumped under” neoliberalism captures the authors’ own primordial differentiation among the policies. There is really no need to brand the review’s constituent papers as ideologically driven, even in the form of a generative mechanism, and by doing so, one is potentially preaching to the liberal democratic choir on the one hand and proffering an insufficient critique of globalized late market capitalism on the other.

 Several specific thematic findings of Glenn et al are persuasive in terms of what works, for whom, and under what circumstances regarding initiatives intended to reduce FS and improve FWB implemented shortly before and during the pandemic. However, one could very well restate the findings using a different ontology than ideology: social welfare policies; labour policies; housing and financial services policies; service provision for health, seniors, childcare and education; community-level and behavioural initiatives. This could make a difference in terms of recommendations and knowledge uptake.

###  2. Real Differences in Policy Options

 In suggesting a pragmatic re-interpretation of Glenn and colleagues’ findings, one could discuss real differences in policy options, not just labelling them neoliberal bad ideas put into practice. Using a neoliberalism label worries us because the underlying drivers of adverse outcomes (including FS) in real-world settings institutionalize their power and privilege over populations through material effects, whether structures or practices. For example, there is potential “misclassification bias” inherent in the “lumping” of included papers under neoliberalism with regards to “radical individualism.” The concept of social in/equity is not inherently at odds with individual in/equity; rather, it takes up the notion that certain forms of governance structure the experience and meaning of equity on a group-level that needs to be accounted for in a relational analysis. To take social equity to be at odds with formulations of liberalism based on individual equity depends on an argument about where “natural rights” (ie, universal, fundamental and inalienable rights) lie. The very use of the outcome of FWB implies individuals seeking to prosper under a market capitalist regime.

###  3. A Non-dialectic Social Equity 

 On the flipside, in Glenn et al, social equity is not an ideology at all (cf Marxism), rather, a set of desired policy outcomes under liberal democratic government, where Guy and McCandless^11^ is presented as the citation and again invoked in Glenn and colleagues’ broadly redistributive argument. But caution is warranted in lumping in redistribution with a universalist (called “holism”) argument, which is now referred to as the “redistributive paradox.”^12^ For instance, emerging scholarship presents the possibility that varied approaches to welfare state provision may achieve social equity.^13^

###  4. Ontological Pluralism 

 Finally, the paper perhaps reminds us that public health has not yet chosen a post-pandemic politics beyond a reinvigoration of focus on identity, as social equity for population health. Post-pandemic public health practice illuminates major concerns about such longstanding health issues as health human resource precarity, the failings of long-term care, and children’s mental, physical and educational well-being—all of which impact FS/FWB absent of community initiatives. To express opposition to a “totalizing” version of neoliberalism is not helpful, nor is a blanket commitment to “social equity” when equity is the desired policy goal. A plural ontology would help us fight for better science and policy, in addition to better theory. There is no neatly packaged pathway for high income nations to proceed upon to intervene on FS/FWB or other markers of health (dis)advantage. Given text examples derive mostly from Canada, the United Kingdom, and Australia—neoliberal globalization cannot be effectively critiqued without situating it in nation-state relations. Detecting horizontal similarities among sub-national socially altern groups in very selected high-income contexts must be done with caution and with consideration of the “real” relational tensions we need to address among diverse equals.^10^

## Conclusion

 We are not the first to call for pragmatism in a moment of crisis and thus we offer our own same old, same old prescription. Whether the ideas of neoliberalism and social equity hold weight (and even reason, or materiality) is besides the point. We, in public health, would probably prefer ourselves to be the champions of social equity on moral grounds. Yet our roots in the practice of improving health and well-being through collective action are what has mattered at the most challenging times in our discipline’s history, including the COVID-19 pandemic.

 Thus, as James^14^ and Dewey^15^ concluded over a hundred years ago, we are calling for greater pragmatism in method, in contrast to more ideological distinction, in the interpretation of the effects of interventions on FWB/FS, and the implications for social justice. And like James, this plea for pragmatism is a “program for more work,” where the pragmatist according to James “*turns away from abstraction and insufficiency, from verbal solutions, from bad a priori reasons, from fixed principles, closed systems, and pretended absolutes and origins. He turns towards concreteness and adequacy, towards facts, towards action and towards power*.”^14^ Ontological pluralism is a necessary pragmatism to improve population health.

## Ethical issues

 Not applicable.

## Competing interests

 Authors declare that they have no competing interests.
